# Severe Esophageal Stricture Caused by Esophageal Candidiasis in a Non-HIV Patient

**DOI:** 10.7759/cureus.46641

**Published:** 2023-10-07

**Authors:** Ahmed H Abdelfattah, Ali M Mahgoub

**Affiliations:** 1 Internal Medicine, University of Kentucky College of Medicine, Lexington, USA; 2 Internal Medicine, and Gastroenterology and Hepatology, Specialized Internal Medicine Hospital, Mansoura University, Mansoura, EGY

**Keywords:** homelessness, immunocompetent adult, adult malnutrition, esophageal stricture, esophageal candidiasis

## Abstract

Esophageal candidiasis (EC) is a common opportunistic infection in immunocompromised individuals, often encountered in situations such as untreated HIV/AIDS or following organ transplantation with immunosuppressant usage. While the main manifestation of esophageal candidiasis is mucosal inflammation, its progression and severe cases may lead to esophageal complications like dysphagia, odynophagia, and weight loss. One of the rare complications is esophageal stricture (ES). Few cases have been reported in the literature to date. Esophageal candidiasis can lead to the formation of ES through chronic inflammation, tissue damage, fibrosis, scarring, and ultimately narrowing of the esophageal lumen. Patients with ES often present with dysphagia, odynophagia, and other symptoms related to impaired swallowing. Esophageal strictures related to EC could seriously affect the patient’s quality of life. Malnutrition and weight loss can be easily encountered in those cases. So, prompt diagnosis and appropriate antifungal therapy are important. Management should include addressing the stricture through endoscopic dilation interventions. Timely recognition of this complication is crucial for improving patient outcomes and quality of life. We present the case of a 46-year-old male with EC complicated by severe ES, dysphagia, and weight loss of more than 30 lbs. The diagnosis was made based on the histopathological examination of the esophageal biopsies.

## Introduction

Esophageal candidiasis (EC) is the most common infectious disease of the esophagus [1,2]. It usually occurs in patients with chronic diseases and immunosuppression [1,2]. In the gastrointestinal (GI) tract, the esophagus is the second most vulnerable to *Candida* infection, after the oropharynx [2]. It can occur in rare cases without known immunosuppression [2]. Many risk factors have been identified to cause EC, like diabetes, corticosteroid usage, cancer, peptic ulcers, and proton pump inhibitor (PPI) usage [2]. Esophageal candidiasis usually presents with symptoms of dysphagia, epigastric discomfort, malnutrition, and the symptoms and signs of the other associated diseases mentioned above. One of the rare complications of EC is esophageal stricture (ES) formation [2].

## Case presentation

The patient was a 46-year-old male who presented to the emergency department (ED) with intractable nausea, vomiting, weight loss, and dehydration. He was found unresponsive before the presentation with hypoglycemia; his blood glucose level was less than 30 mg/dl. The patient responded to intravenous glucose and thiamine. The patient has poor social support and housing instability. The past medical history was significant for substance use disorder, for which he is currently on buprenorphine or naloxone; chronic hepatitis C infection (but without signs or symptoms of hepatic decompensation); and malnutrition due to his poor social situation.

The patient stated that he had been having epigastric pain for a while but could not remember exactly when it started. This pain was aggravated by food intake, and nothing could relieve it. The patient also mentioned that he had persistent nausea and vomiting, almost daily. His oral intake has been poor in the last few months, to the extent that he lost more than 30 lbs the previous year. The patient had multiple ED visits in the past; one of them was a few months prior to this presentation with a concern for gastrointestinal (GI) bleeding. At that time, esophagogastroduodenoscopy (EGD) was done on an urgent basis and completed successfully. It was significant for severe grade D esophagitis, with a pale appearance of the gastric mucosa and normal duodenum. He was treated with a PPI course lasting eight weeks. During that admission, the physical examination was significant for ill appearance, cachexia, and epigastric tenderness, but no oral thrush was present back then.

**Figure 1:**
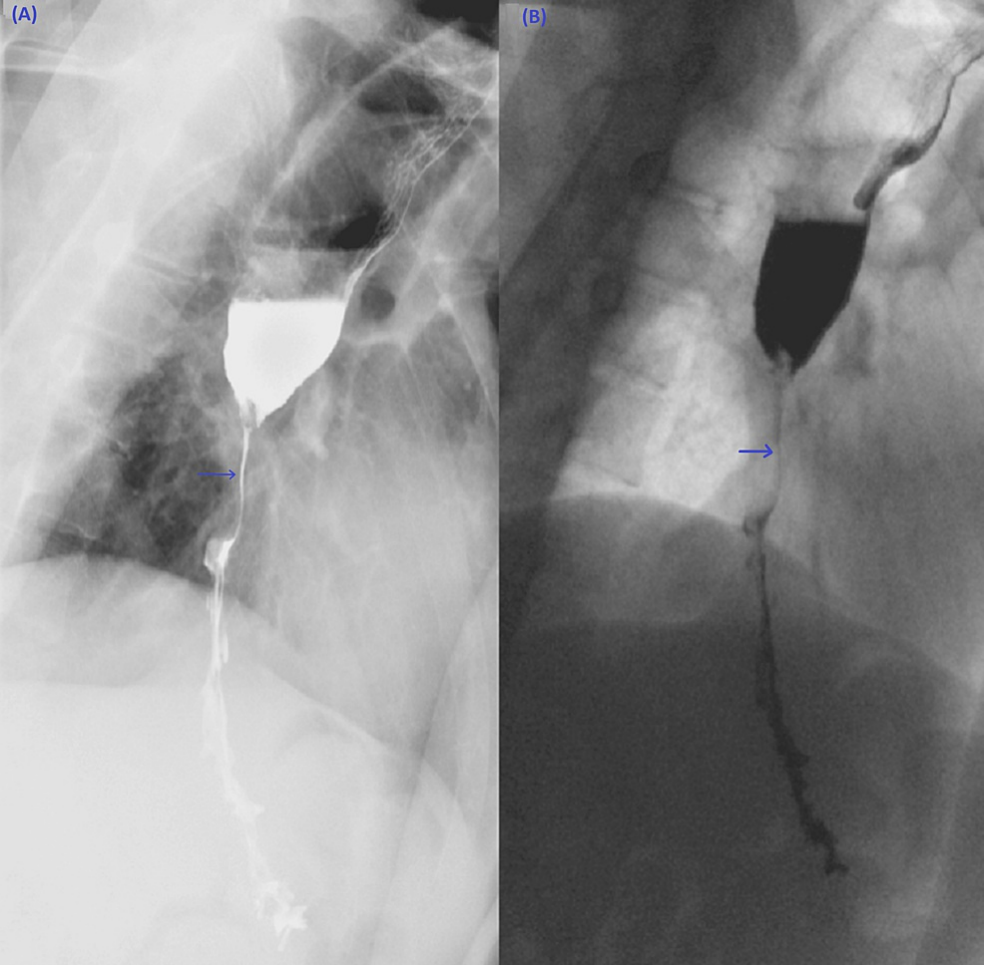
Single contrast barium fluoroscopic evaluation of the upper GI Blue arrows in A and B show severe narrowing of the distal third of the esophagus with marked upstream dilation. GI: Gastrointestinal

The patient was admitted to the medical floor for further investigations and workups. Laboratory investigations were significant for hyponatremia of 132 mmol/L, hypochloraemia of 94 mmol/L, normocytic normochromic anemia with hemoglobin (HB) of 9.3 g/dL and hematocrit (Hct) of 29.5%, and high aspartate aminotransferase (AST) and alanine transaminase (ALT) of 424 and 217 U/L, respectively. Despite the supportive and conservative management of the patient's symptoms, there was no improvement in the nausea and vomiting. So, a barium fluoroscopy swallow study was done and showed severe narrowing of the distal third of the esophagus with marked upstream dilation (Figure 1).

**Figure 2:**
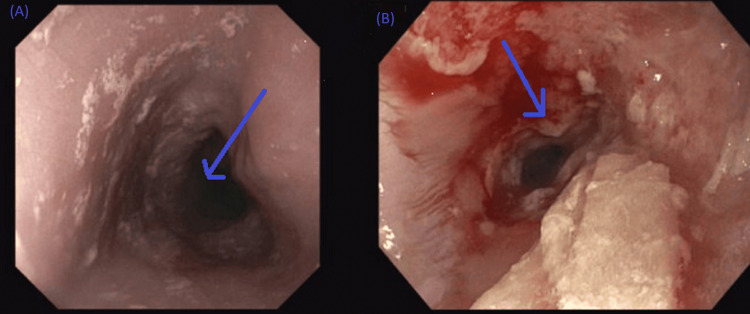
EGD image of the lower end of the esophagus Blue arrows in A and B show severely inflamed ES (not traversable) in the lower third of the esophagus. EGD: Esophagogastroduodenoscopy, ES: Esophageal stricture

A CT of the chest showed no evidence of extrinsic compression of the esophagus. The GI team was consulted about the possible need for an EGD and an esophageal biopsy. The EGD showed severe generalized edematous, fissured, friable, and ulcerated mucosa with whitish exudate and plaques in the esophagus. However, EGD was not completed because of the narrowing and the ES present at the lower end of the esophagus, which hindered further advancement of the scope (Figure 2).

Multiple biopsies were taken, and EC was diagnosed based on the histopathological results. The patient tested negative for HIV. The decision was made to start him on antifungal therapy for three weeks, then to repeat EGD for possible dilatation after completion of the antifungal treatment. The IV micafungin was used initially. With the start of the IV treatment, the patient reported feeling better and was switched to oral fluconazole after two weeks of IV micafungin, with a plan to follow up with the GI team for a repeat EGD for dilatation. The diet was advanced gradually, and the patient tolerated it well. The diagnosis of esophageal stricture related to candidiasis infection was established based on the histopathological findings and the patient's response.

## Discussion

*Candida albicans* is part of the usual flora of the oropharynx and lower GI tract and is normally not pathogenic. It is the most common cause of esophageal infections. There are numerous *Candida *species, including *Candida tropicalis* and *Candia guilliermondii*, that have been linked with esophageal infection, but *C. albicans* account for the great majority of these infections [3]. Although EC rarely occurs without a clear underlying cause, even in immunocompetent hosts, a predisposing factor should be assumed [4]. Risk factors for* Candida* infections include but are not limited to HIV infection, malignancy, chronic alcoholism, diabetes, prolonged use of antibiotics or immunosuppressive drugs, or esophageal dysmotility disorders like achalasia or scleroderma [5].

Esophageal candidiasis is distinguished by the presence of white pseudomembranous or plaque-like lesions attached to the esophageal mucosa during endoscopy. The frequency of EC encountered at the EGD ranges from 1% to 8%, with decreased frequency after the introduction of the newer antifungals in the 1990s [6]. Moreover, double-contrast barium esophagography can aid in the diagnosis, but it is less sensitive compared to endoscopy [2,6]. Chronic mucosal inflammation usually involves the lamina propria initially and then progresses to transmural inflammation, fibrosis, scarring, and luminal stricture. Rarely, intramural pseudodiverticulum can occur from the dilatation of submucosal glands [5].

The typical symptoms and findings in EC and strictures are dysphagia and odynophagia, in addition to white plaques that can be found orally and endoscopically that can’t be irrigated by water [2,5,6]. In rare cases, mass-like lesions and ES can be encountered as a complication of EC. There are a few cases reported in the literature regarding candidiasis-associated ES. Most of these cases are seen in patients with concurrent diseases such as diabetes, malignancies, leukemia, and glycogen storage disease [2,5,6].

Candidiasis-associated ES is much more infrequent in immunocompetent patients, like our patient [5]. The only risk factor that we can blame in our case is malnutrition, which resulted from the poor social situation and housing instability, and to the best of our knowledge, it was not reported before. These strictures are usually encountered distally in most cases, with only a minority of cases having proximal ES occupying the upper or middle thirds [5].

Esophageal candidiasis is treated with antifungal antibiotics, which can be given orally or intravenously if the dysphagia is severe for 14 to 21 days [2]. Management of candidiasis-associated ES is usually challenging, with most of the cases needing to be treated endoscopically with dilatation, with only one case reported in the literature to be improved after two weeks of antifungal treatment [6,7]. In another case, the ES was encountered in an immunocompetent patient who experienced a recurrence of EC despite antifungal treatment and required repeated endoscopic interventions to dilate the stricture [5]. The ES can become devastating if the treatment is delayed or inadequate [7]. So, prompt clinical diagnosis and management are very important.

## Conclusions

Candidiasis-associated ES is a rare complication that can be encountered even without an apparent immunocompromising state. This case highlights the importance of considering candidiasis in the differential diagnosis of ES. Clinicians should be aware that prompt clinical diagnosis and management are crucial, as the stricture can be debilitating and have massive effects on patient outcomes and quality of life.
